# Feasibility and Acceptability of ‘Opt-In’ Referrals for Stop Smoking Support in Pregnancy

**DOI:** 10.3390/ijerph16081358

**Published:** 2019-04-16

**Authors:** Katarzyna Anna Campbell, Sophie Orton, Katharine Bowker, Sue Cooper, Tim Coleman

**Affiliations:** Division of Primary Care, University of Nottingham, Tower Building, University Park, Nottingham NG7 2RD, UK; Sophie.orton@nottingham.ac.uk (S.O.); katharine.bowker@nottingham.ac.uk (K.B.); sue.cooper@nottingham.ac.uk (S.C.); tim.coleman@nottingham.ac.uk (T.C.)

**Keywords:** smoking cessation, pregnancy, ‘opt-in’ referrals

## Abstract

Background: International guidelines recommend that following an early-pregnancy ‘opt-out’ referral for smoking cessation support, pregnant women who smoke should also be offered referrals at subsequent antenatal appointments (‘opt-in’ referrals). We assessed feasibility and acceptability of introducing ‘opt-in’ self-referral forms to stop smoking services (SSS) in antenatal clinics. Method: A ‘before–after’ service evaluation and qualitative interviews. ‘Opt-in’ self-referral forms were distributed by reception staff to women attending antenatal ultrasound appointments. We collected hospital/SSS data for the study period and a comparison period 12 months prior. Reception staff were interviewed and data analyzed thematically. Results: Over 6500 women entered antenatal care in each period; ~15% smoked and ~50% of those who smoked were referred to SSS at their first appointment. In the study period, 17.4% of women completed ‘opt-in’ forms. Of these 17.3% smoked, and 23.1% of those who smoked requested a referral. The staff thought new procedures had minimal impact on workload, but were easy to forget. They believed the pathway would be better delivered by midwifery staff, with additional information/advice to improve engagement. Conclusions: ‘Opt-in’ referrals in later pregnancy result in significant numbers of women who smoke indicating interest in smoking cessation support. Additional training and support is necessary to motivate reception staff to oversee self-referral pen-and-paper procedures effectively.

## 1. Introduction

Smoking in pregnancy remains a concern for healthcare systems worldwide. In 2015, in the European Region, 8.1% of women were found to smoke in pregnancy, with rates as high as 38.4% in Ireland and 29.4% in Bulgaria [1]. In the Region of the Americas the rates were just under 6% [1]. In 2018, in England, approximately 11% of women smoke throughout their pregnancy [2]. Compared to women who do not smoke in pregnancy, smoking mothers have an increased risk of fetal and infant morbidity and mortality [3]. The World Health Organization (WHO) and UK National Institute for Health and Clinical Excellence (NICE) recommend that all pregnant women should be asked about their smoking status early on in their pregnancy, and that their smoking status should be assessed at this point using carbon monoxide (CO) testing. Subsequently, unless they specifically decline, all women who smoke should be referred to specialist services for smoking cessation support (‘opt-out’ referrals) [4,5]. This approach was found to successfully engage women and have a significant positive impact on cessation [6,7]; it is, however, also known that up to 75% of women referred to stop smoking services (SSS) do not take up these appointments and of those who do, up to 35% do not quit [6,8]. Some of the women who manage to quit in pregnancy relapse to smoking before the end of their pregnancy or shortly after childbirth. Therefore, guidelines also recommend that at all subsequent antenatal appointments women should be asked if they took up their referral as well as if they managed to quit smoking. If they did not, they should be asked if they are interested in stopping smoking and offered another referral for cessation support (‘opt-in’ referral) [4]. 

The aim of this study was to assess the feasibility and acceptability of introducing simple ‘opt-in’ self-referral methods later in antenatal care and after women had already experienced ‘opt-out’ referrals. Feasibility, or how achievable the introduction of this method was in practice, was assessed using numbers of forms handed out to patients, forms completed, and referrals generated via this method. Acceptability of these procedures to staff was assessed via semi-structured interviews in which they were asked to describe their views, opinions and experiences with delivering the ‘opt-in’ referrals to pregnant women. We also report relevant smoking cessation outcomes; however, in addition to aims stated above, another study aim was to test if this referral method could increase referrals to SSS later in pregnancy.

## 2. Materials and Methods

### 2.1. Study Design

**Figure 1:**
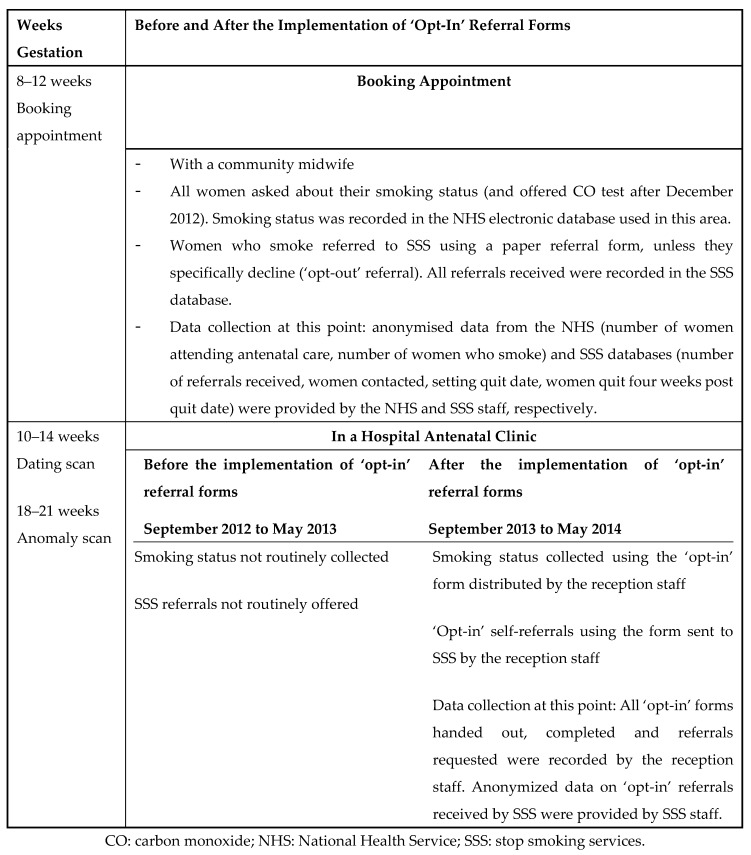
Smoking cessation practices and study data collection at key antenatal appointments in the periods before and after implementation of the ‘opt-in’ referral forms.

This was a multi-methods study with a ‘before–after’ service evaluation design, set in two antenatal ultrasound scan departments in one National Health Service (NHS) Trust in the UK. We collected hospital process and outcome data during a nine month study period (the ‘after’ period) and a nine month comparison period one year earlier (the ‘before’ period). Smoking cessation practices at key antenatal appointments and data collection procedures in both study periods are presented in Figure 1. In addition to the quantitative evaluation of feasibility, qualitative interviews with staff involved in the delivery of the ‘opt-in’ referrals pathway were conducted post-study to evaluate acceptability of this referral method.

In the ‘before’ period (1 September 2012 to 31 May 2013), all women, as part of their routine care, were asked about their smoking during their first antenatal (‘booking’) appointment with community midwives at 8–12 weeks gestation. Pregnant women who smoke or recently quit were referred to SSS unless they specifically declined (‘opt-out’ referral pathway). Women were asked about their smoking at subsequent midwife appointments, but not during antenatal scan appointments; further referrals were not routinely offered throughout the rest of pregnancy. Following the introduction of the NICE guidance [4] suggesting that all women in the UK should be asked about their smoking, and that carbon monoxide (CO) screening should be used to validate their smoking status, from December 2012 the Trust began using CO monitors at booking, to identify women who smoke and offering ‘opt-out’ referrals to SSS at this point. This procedure was not well-established in this Trust until later on in 2013.

In the ‘after’ period (1 September 2013 to 31 May 2014), in addition to the routine care outlined above, ‘opt-in’ referral forms (see Supplementary File S1) were offered at the antenatal clinics’ reception desks to all women attending for ‘dating’ (10–14 weeks gestation) and ‘anomaly’ (18–21 weeks gestation) ultrasound scan appointments, regardless of their smoking status. Women were asked to take a form to the waiting room, where they had the time to read the information and complete the form. There was no designated private room/area for this task. The form followed WHO [5] and NICE [4] recommendations and briefly explained the benefits of quitting in pregnancy and SSS support available and asked women about their smoking status. Women who reported smoking in pregnancy were asked if they were already receiving support, and those who did not, were offered a referral to SSS. Women interested in receiving support were asked to give contact details so that the SSS could contact them.

The ‘opt-in’ referral form was piloted and discussed with 30 pregnant women in one of the antenatal clinics, to ensure this form was appropriate and understandable for the target population. Amendments were made following the feedback, to further improve clarity of the form. KAC was introduced to the reception staff by the clinic manager; she explained the NICE guidelines for smoking cessation in pregnancy [4] and introduced the form. She explained expectations regarding handing out, collecting and sending completed forms to SSS. She stressed the forms were self-explanatory and staff only had to ask women to read, complete and return forms back to the reception. Written instructions for staff, forms, pens and envelopes were provided. KAC also visited reception areas monthly to check if staff had any problems or issues with the procedures, to replenish supplies of the forms and to collect information on the progress of the study. Staff were asked to hand forms out to all women attending scan appointments and, where referral was requested, to send this via secure mail to the SSS, which was situated in another location in the city centre. After receiving the forms, SSS staff attempted telephone contact with women up to three times and also sent a letter offering help; those reached were encouraged to set quit dates and were offered standard SSS support.

### 2.2. Data Collection and Analysis

All data were anonymized by hospital and SSS staff before being passed on to the research team. From hospital and SSS records we obtained numbers of women ‘booked’ for antenatal care, their smoking status, numbers routinely referred for cessation support at booking appointment, as well as whether or not quit dates were set and cessation for up to 4 weeks was achieved. Routine data were analyzed descriptively in Stata V.13.1 (StataCorp LP, College Station, TX, USA); where presented, 95% confidence intervals (CI) for proportions were calculated using Wilson score method [9] and 95% CIs for the difference between two proportions were estimated based on the Newcombe-Wilson method without continuity correction [10].

In July 2014, semi-structured interviews were conducted with reception staff. We invited all six staff members involved in ‘opt-in’ referrals to take part in the interviews; three refused due to time constraints and three consented and were interviewed face-to-face by SO (an author). SO is a female researcher who at the time of data collection was a PhD student, with an MSc in Health Psychology and experience of qualitative research. She worked in the field of smoking and pregnancy for a number of years and some of the staff were familiar with her from previous research projects over the years, which was helpful in building rapport. A semi-structured interview schedule was used to ensure all participants were asked about their views and experiences with the ‘opt-in’ pathway in a similar manner and to reduce interviewer bias. The interview schedule was designed by KAC, the lead researcher on this project who was familiar with the study procedures, with input and guidance from the other authors. The guide was pilot tested with another team member. The schedule is available as a Supplementary File S2.

SO was not involved in the project prior to interviews, and therefore, it was considered more appropriate for her to conduct interviews, as she would have no preconceived ideas about the project, including about its acceptability to staff. As KAC had been directly involved in setting up the ‘opt-in’ project, liaising with staff and providing them with the initial training and support it was considered possible that her close involvement could potentially affect the participants’ readiness to speak openly about any negative views they had of the project.

All participants were female staff working in the antenatal clinic receptions, all were non-smokers. Interviews were audio-recorded and transcribed verbatim, using transcription services. Each interview lasted approximately 30 minutes. Data were analyzed thematically following a simplified Braun & Clarke’s method [11]. Analysis was pragmatic, and aimed to identify themes that could explain how the participants felt about the introduction of self-referral forms within the reception environment. The data were analyzed inductively by KAC, who read and re-read the transcripts, noting concepts of interest. These were then discussed with SO, and the two authors combined these concepts into key themes. The dataset was then re-read to establish that the themes were representative of the views of all participants. To improve the quality of reporting of the qualitative research, we have followed the consolidated criteria for reporting qualitative research (COREQ) [12].

### 2.3. Ethical Approval

Routine antenatal care was changed by the NHS Trust for all pregnant women attending antenatal care; therefore, the project was categorized as a ‘service evaluation’ and as such did not require ethical approval. The project was registered with NHS Trust as an audit (reference number 13-022C). Only anonymized data were collected from routine sources, thus consent from individuals was deemed unnecessary. Written consent from interview participants was obtained prior to the interviews. 

## 3. Results

Similar numbers of women were booked for antenatal care in both ‘before’ and ‘after’ periods (Table 1), however more women who smoked were identified at booking in the ‘after’ period (*p* = 0.0356). In both periods, around half of those who smoked were referred for SSS support at booking (54% 537/993 ‘before’ and 45% 497/1046 ‘after’).

In the ‘after’ period, 17.4% (95% CI: 16.5 to 18.3) of booked women completed the ‘opt-in’ form (Table 1). Three percent (95% CI: 2.6 to 3.4) of booked women reported smoking via the ‘opt-in’ form; this amounts to 17.3% of those who completed the form. Less than 1% (0.7%, 95% CI: 0.5 to 0.9) of women booked requested a referral; this amounts to 23.1% of women who reported smoking via the ‘opt-in’ form. SSS managed to contact seven women who requested support via ‘opt-in’ form, but only one of these engaged with the service and set a quit date. The remaining 39 women were either uncontactable or refused support.

Overall, when ‘opt-out’ and ‘opt-in’ referrals were combined, there were no differences between periods in numbers of women referred, setting quit dates or reporting cessation (Table 1). 

### Interviews

The following three themes relating to the acceptability of the ‘opt-in’ referral forms were identified: impact on workload, views on ‘opt-in’ procedures and confidence.

*Impact on workload*: Interviewees reported that the reception was very busy, and pressured. While all agreed the ‘opt-in’ pathway had minimal impact on workload, requiring approximately 10 minutes/day to handle the forms, they admitted it was easy to forget amidst their hectic schedule. They understood the task, but they perceived it as ‘a minor inconvenience’ and part of a research project, rather than a way to help women quit smoking.

P3: *It was just something I was doing. I was obviously doing another job so it wasn’t extra work as such, it was just no, it didn’t feel like extra work it was just different work if you like, something different to just remember to do.*

P2: *The impact [of introducing the ‘opt-in’ procedures]… massively busy clinics, people on one side of you, people on the other, asking you will you do this, will you do that? It’s an added extra thing to give out, so sometimes it will be forgotten. I think everybody who’s handed them out would say there will be a few times when they’ve forgotten to hand them out.*

*Views on ‘opt-in’ procedures*: Interviewees agreed the reception is an appropriate place to capture the majority of women attending scan appointments, but not private enough to discuss smoking in detail. They felt pen-and-paper forms were acceptable in this environment, as they offered greater level of privacy than a face-to-face discussion. Nevertheless, they felt forms were not engaging enough and easy to ignore or to result in misrepresentation of smoking status.

P3: *I think the reception is the right place [to hand out the forms], but I do feel that, I mean that’s the place sort of where we can hand them out or take them in, but I think that it’s somebody that is actually involved in it that should be explaining it for the patients. Because basically I were just handing out a piece of paper and that was it, you know, I didn’t, wasn’t involved in it as such, so I didn’t know a vast amount about it. I mean people didn’t really ask, if they wanted to fill it in, they filled it in, they gave it me back, I think (…) it would have probably got more people interested if there was somebody actually there, you know, talking to the patients*

P2: *Well obviously if you hand something out to somebody, you rely on them to fill it in and give it back to you. Whereas if you’re communicating verbally with somebody then you know you’re more assured of having the responses that you want, because it’s more difficult to not respond if somebody’s talking to you.*

*Confidence:* Interviewees felt that addressing smoking was not part of their role. They were unsure of the effects of smoking on the fetus and while they were aware of SSS, they felt they did not have enough knowledge about smoking in pregnancy or SSS to confidently discuss referrals.

I: *And how do you feel about smoking in pregnancy, what are your opinions?*

P1: *Hmm, obviously if they can stop, well like I say I don’t know what it’s… health wise what it does to the fetus. Just if it does harm the fetus/baby then yeah I think they ought to stop.*

P3: *I’ve heard of [Stop Smoking Services]. I’ve heard things, emails in hospital, you know for staff about stopping smoking. Not taken a lot of interest because as I say I’ve never smoked. So I don’t, but yes I have heard of it.*

They felt they were possibly not the best placed staff to discuss smoking with patients and suggested that especially trained midwifes or auxiliary maternity staff working in private rooms would be more suitable to offer referrals. Some were worried that bringing up smoking with pregnant women might cause upset.

P1: *I suppose we’re not trained to give advice, but I suppose if I’d have said as a receptionist if I’d have been asked to give advice I wouldn’t have done, because I don’t think I would have got a nice response back from these, any ladies because some can be a bit off with you and no I don’t, no I wouldn’t have liked to have given advice out. And as a clerical officer/receptionist I wouldn’t like to have done it anyway.*

*(…)I think I’d rather leave that up to the midwives to do that, because some of [the women] can get offended and I don’t feel like being shouted at, at the reception desk.*

They were also unconvinced about the potential effectiveness of offering ‘opt-in’ referrals at ultrasound appointments and some acknowledged that should the procedures be followed by all the reception staff at all times, more women who want to quit might have been captured by these procedures:

P3: *No I think for those that filled it in it went well, but I think there probably could have been more people fill it in. Had we caught more patients or there’d been somebody there constantly. Or at least at times when I wasn’t there, because I know that when I had days off or weeks off I don’t think any were offered out.*

## 4. Discussion

To our knowledge, this is the first evaluation of offering ‘opt-in’ referrals for smoking cessation support after ‘opt-out’ referrals had been made earlier in pregnancy. Self-completed referral forms distributed at antenatal ultrasound appointments engaged 17.4% of women attending antenatal care and resulted in 23.1% of self-identified smokers requesting referral for smoking cessation support. Clinic reception staff, however, felt insufficiently prepared and not best placed for giving out smoking cessation information and referral forms, and fewer than 30% of women who we estimated to attend ultrasound appointments received them.

This study used a ‘before–after’ evaluation with qualitative investigation of staff views to assess feasibility and acceptability of introducing simple self-reported ‘opt-in’ referral forms delivered by reception staff. Therefore, the non-randomized design makes it difficult to attribute causality and is a limitation of this study. Another limitation is the fact that we only interviewed half of the staff involved, and the sample was small. Although the views were largely unanimous and helped us understand issues which receptionists considered important as well as the potential ways to improve ‘opt-in’ procedures in the future, it is possible that staff members who agreed to be interviewed had different opinions on the ‘opt-in’ procedures to those who declined. Additionally, as our work was conducted in only one hospital Trust the generalizability of our findings may be reduced. However, as little is currently known about the views of reception staff on engaging in smoking cessation referral procedures, this study offers an interesting starting point for future investigations. In addition, the study Trust had a higher rate of smoking at delivery than the concurrent English average and findings might not be generalizable to hospitals with substantially lower rates. 

In both study periods, only around 50% of identified women who smoke were referred to SSS at booking, despite ‘opt-out’ referrals being operational and well-established in this area. Therefore, many women offered later ‘opt-in’ referrals might not have been aware of the available support. Furthermore, fewer ‘opt-in’ referrals might have been generated if more women had received ‘opt-out’ referrals at booking. Half-way through the ‘before’ period the Trust introduced CO screening, which became operational in the ‘after’ period and the statistically significant increase in the percentage of women who smoke in pregnancy identified at booking between the two periods might be attributable to this service development. This change followed a recent introduction of the NICE guidance [4], which influenced how smoking was addressed across the country, likely improving the practices of identifying and referring pregnant women who smoke early on in pregnancy. Therefore, our findings may be generalizable to other hospitals in which robust methods of identifying pregnant women who smoke at booking are used.

A study strength is the use of both quantitative and qualitative methodology to evaluate how practical the introduction of ‘opt-in’ referral methods in antenatal clinic reception would be. Qualitative findings helped explain the quantitative results, particularly the relatively low numbers of women who completed the ‘opt-in’ referral forms. Interviews with staff revealed that although they found the ‘opt-in’ procedures easy to follow, they did not perceive them as a priority, possibly because of their insufficient knowledge on the harms of smoking as well as the potential support SSS could offer. Potentially, with extra support and training on harms of smoking as well as the stop smoking services, receptionists could be more motivated and confident about distributing forms. In previous studies, appropriately trained health care assistants were found to be able to competently deliver ‘opt-out’ referrals involving CO testing [6,7]. Reception staff in the current study, however, were not confident or motivated to deliver a much more straightforward task of distributing referral forms. They were also concerned that any conversations about smoking with women should be conducted face-to-face to improve engagement. They also felt that, as these conversations relate to health, they should be carried out by medical professionals rather than the administrative staff, in private to ensure confidentiality. With specific training on cessation support and reassurance that no public conversations about women’s smoking status would be needed unless women wanted to discuss it, one could envisage receptionists being enabled to distribute referral forms much more systematically. On the other hand, we acknowledge that helping pregnant women who smoke achieve cessation is a complex and difficult process and a more engaging and tailored approach might be necessary to capture and support them at later stages of pregnancy, if they did not engage with SSS or quit at this point.

‘Opt-in’ referral forms appeared more acceptable to women attending clinics than to the receptionists as 65% of those who took the form completed it (1150/1800) and a quarter of self-reported smokers sought referral. However, of these ‘support-seeking’ women, only one engaged with the SSS and set a quit date with SSS support. In most cases, the SSS were unable to engage women, because they did not answer the phone or declined support; six women accepted support but did not attend the appointment. In future evaluations it would be useful to monitor how women are engaged in SSS support after ‘opt-in’ referrals are received and the reasons why some change their mind about receiving support. Future research should also aim to understand the gap between women’s intention expressed through the ‘opt-in’ referral form and their willingness to engage with cessation support, in order to improve engagement and quitting behaviors.

## 5. Conclusions

It is evident that following ‘opt-out’ referrals in early pregnancy, many pregnant women are still interested in being supported to stop smoking and are willing to ask for support. This supports the need for developing systematic ‘opt-in’ referral procedures later on in pregnancy. Assuming the process piloted in this study could be made more acceptable to reception staff by targeted training and support, self-complete, ‘opt-in’ referral forms could be a cheap, simple means for increasing referrals of pregnant women for smoking cessation support. It is, however, possible that a more involved approach might be needed to successfully engage pregnant women who smoke and who refused support early on in pregnancy.

## Figures and Tables

**Table 1:** Referrals of pregnant smokers received by stop smoking services and outcomes before and after implementation of the ‘opt-in’ referral pathway.

	Before			After			Difference in Proportions (95% CI)
	N	% of Women Attending Booking **	95% CI	N	% of Women Attending Booking **	95% CI	
Women attending booking appointment	6825			6613			
‘Opt-in’ forms distributed	NA			1800			
‘Opt-in’ forms completed	NA			1150	17.4	16.5 to 18.3	
Women who smoke:							
At booking	993	14.5	13.7 to 15.3	1046	15.8	15.0 to 16.7	1.3 (0.1 to2.5) *p* = 0.0356
Via ‘opt-in’	NA			199	3.0	2.6 to 3.4	
**Total**	993	14.5	13.7 to 15.3	NA *			
Referrals							
At booking	537	7.9	7.3 to 8.5	479	7.2	6.6 to 7.9	0.7 (−1.2 to 1.6); ns
Via ‘opt-in’	NA			46	0.7	0.5 to 0.9	
**Total**	537	7.9	7.3 to 8.5	525	7.9	7.3 to 8.6	0.0 (−0.9 to 0.9); ns
Set QD							
At boking	157	2.3	2.0 to 2.7	160	2.4	2.1 to 2.8	0.1 (−0.4 to 0.6); ns
Via ‘opt-in’	NA			1	0.01	0.002 to 0.08	
**Total**	157	2.3	2.0 to 2.7	161	2.4	2.1 to 2.8	0.0 (−0.4 to 0.6); ns
Quit 4 weeks							
At booking	136	2.0	1.7 to 2.4	131	2.0	1.7 to 2.3	0.0 (−0.5 to 0.5); ns
Via ‘opt-in’	NA			1	0.01	0.002 to 0.08	
**Total**	136	2.0	1.7 to 2.4	132	2.0	1.7 to 2.3	0.0 (−0.5 to 0.5); ns

* We did not combine smokers identified at booking and via ‘opt-in’, as some of these women might have been identified on both occasions. ** We report outcomes within pregnant women rather than within smokers because the method of identifying smokers varied in the two time periods and, hence, the population of pregnant women provided a denominator that was not prone to vary between. NA: not applicable; ns: non-significant.

## Data Availability

All data are available on request from the corresponding author.

## Supplementary Materials

The following are available online at https://www.mdpi.com/1660-4601/16/8/1358/s1, Supplementary File S1: ‘Opt-in’ referral form; Supplementary File S2: Interview schedule.
