# Supplementary material for: Feasibility and Acceptability of ‘Opt-In’ Referrals for Stop Smoking Support in Pregnancy
Source: Int J Environ Res Public Health. 2019 Apr 16;16(8):1358. doi: 10.3390/ijerph16081358 (PMC6518207; doi:10.3390/ijerph16081358)
Supplement: Supplementary file 1 [file ijerph-16-01358-s001.zip › Supp File 1 OptInForm.pdf]

Supplementary File 1. 'Opt-in' referral form.

Nottingham University Hospitals NHS
NHS Trust

Opt-in referral to NHS Stop Smoking Services

Congratulations on your pregnancy

We are looking at new ways of improving health and wellbeing of pregnant women and their babies. We are currently inviting ALL women who come to antenatal scan appointments to answer a few questions about smoking and can offer referrals to local NHS Stop Smoking Services.

Your answers are confidential and will only be used to determine if you wish to receive stop smoking support. If you do want support, your details will be passed on to your local NHS Stop Smoking Services.

**Q1. Have you smoked any cigarettes or tobacco in the last four weeks?**<br><br>Please answer 'YES' even if you have had only one cigarette.	**YES** ☐<br>**NO** ☐
If you have answered '**YES**' to Q1 above, **please continue to the next question**.<br>If you have answered '**NO**' to Q1 above, you may stop here. Please hand the form back to the Reception. Thank you for your time.	
**Q2. Are you currently getting any support from the NHS Stop Smoking Services, such as New Leaf?**	**YES** ☐<br>**NO** ☐
If you have answered '**NO**' to Q2 above, **please continue reading**.<br>If you have answered '**YES**' to Q2 above, you may stop here. Please hand the form back to the Reception. Thank you for your time.	

Please read the following information carefully:

Pregnancy is a perfect time to stop smoking because protecting your baby from the harmful effects of tobacco smoke is one of the best things you can do to give them a healthy start in life.

Stopping smoking can feel difficult on your own. Getting extra support from a trained stop smoking advisor can make a big difference and help you quit, and stay quit, for good.

Your local NHS Stop Smoking Service can offer you one-to-one, phone and text support, as well as a range of free nicotine replacement products.

To find out how to get free support in stopping smoking,

please turn over and continue

**Q3. Would you like your local NHS Stop Smoking pregnancy advisor to contact you to discuss the support they can offer to help you stop smoking?**	**YES** ☐<br>**NO** ☐	

If you have answered 'YES' to Q3 above, and
<u>if you agree for your details to be sent to you local NHS Stop Smoking Services</u>,
please complete the form below.

Name:			
Telephone	Day:<br>Mobile:	Evening:	
Best time to call you:			
Home Address:			
Email address:			
Your GP Surgery:			
Date of Birth:		Due Date:	
Your signature:		Date:	

Office use only		
ID:	Date completed:	Date passed to New Leaf:

Thank you. Please tear here, return the top part of this form to the Reception and keep the information below.

Why do we need your telephone number, email and address?

Your details will be passed on to the NHS Stop Smoking Services so that they can contact you. If they have problems reaching you by phone, they may write to you or email you.

Who will have access to your details?

Only designated staff at the hospital who are responsible for making referrals to the NHS Stop Smoking Services and the NHS Stop Smoking specialists will have access to your personal details.

Contact the NHS Stop Smoking Services

If you have answered 'NO' to Q3 you can change your mind at any point. If you decide you would like to get some support to stop smoking, you can contact the NHS Stop Smoking Services and leave your details – an adviser will call you back.

Organisation *Telephone number here*
