# Supplementary material for: Feasibility and Acceptability of ‘Opt-In’ Referrals for Stop Smoking Support in Pregnancy
Source: Int J Environ Res Public Health. 2019 Apr 16;16(8):1358. doi: 10.3390/ijerph16081358 (PMC6518207; doi:10.3390/ijerph16081358)
Supplement: Supplementary file 1 [file ijerph-16-01358-s001.zip › Supp File 2 Interview schedule.pdf]

Supplementary File 2. Interview schedule

Opt-in staff interviews

Aims

1. To explore staff's views on smoking and smoking cessation in pregnancy
2. To explore staff's views on the process of implementation of the opt-in project, including barriers and facilitators.
3. To explore staff's views on effectiveness of the referral pathway.

Introduction

Name, affiliation, explain this is to learn about their views on the process, to evaluate and improve the implementation in the future; consent to take part, to record, to use quotes, anonymous, confidential, not a test, honest views and opinions.

Questions

(a) Role of receptionists
What is your role in the antenatal clinic/antenatal scan reception? What are your main duties; extra duties (e.g. relating to smoking advice? Research?); workload; support?

(b) Understanding of the project and its merits:
The stop smoking in pregnancy project (explain or remind them, if necessary) was piloted in the clinic for the last 9 months
(Remind them of the green forms.)
What do you understand the opt-in project was about?
What, in your opinion, was the point of it?

(c) Views on smoking and smoking cessation in pregnancy:
How do you feel about smoking during pregnancy?

Government guidelines recommend that women should be asked about their smoking at every opportunity during their pregnancy, offered a brief smoking advice and a chance to request a referral to SSS.

Have you heard about the local stop smoking services before the project? What do you know about them?
What do you think about asking women at all antenatal appointments about their smoking habits and offering them referrals to SSS?
What outcomes can this potentially bring? (Impact on women and their smoking behaviours, impact on the antenatal services staff who have to include smoking advice in their routine care;
How do you think it makes the women feel?

Views on implementation of the pathway:

(d) Views on where/by whom implemented:
Do you think that handing out the referral forms at the reception was appropriate? Why?
Can you think of a better place/more appropriate person(s) within the antenatal clinic to offer the ladies a chance for an opt-in referral? Why?

(e) Views on format – pen and paper:
What do you think about asking the ladies about their smoking/offering referrals by using a pen and paper, self-completed forms? Benefits? Disadvantages? Do you think it works? Why?
What in your opinion would be a more effective way of approaching them about smoking at this point? Why?

(f) Views on incorporating opt-in pathway into their daily routine:
What were your tasks in relation to the opt-in study? / What were you asked to do on a daily basis for this project? How did you feel when you were asked to do this? Did you feel adequately prepared?
Handing out the forms – did you hand them out or were they on the desk for people to help themselves? What did you say to the ladies who arrived at your desk about these forms?
How did the opt-in pathway fit into your daily routine?
How much time on average did it take you to deal with these forms?
Were there any tasks you found particularly difficult to take on? Why?
What were the main barriers to carrying out these tasks related to the pathway?
What, if anything, helped you carry out these tasks?
What impact did the pathway have on your workload?
What could have been done to make it easier to incorporate the pathway into your daily routine?
Did your initial feelings about the tasks alter once you started doing them? In what way did they change? Why do you think your feelings changed?

(g) Women's reactions to the pathway
When you handed out the forms to the ladies, what responses did you get? What did they say/do? How did they react? Did many take the form? What was the reasoning for not taking the form? Did women ask any questions? How did you handle these?

(h) What could be the potential outcomes of offering the ladies a Stop Smoking Services referral form at all antenatal appointments? Benefits? Harms?

(i) Perceived impact of the pathway; on the women; on the reception staff;

(j) Future directions – continuation; improvements; changes.
